# Novel grading system for quantification of cystic macular lesions in Usher syndrome

**DOI:** 10.1186/s13023-015-0372-0

**Published:** 2015-12-10

**Authors:** Ieva Sliesoraityte, Tunde Peto, Saddek Mohand-Said, Jose Alain Sahel

**Affiliations:** Centre Hospitalier National d’Ophtalmologie des Quinze-Vingts, DHU Sight Restore, INSERM-DHOS CIC 1243, 28 rue de Charenton, 75012 Paris, France; NIHR Biomedical Research Centre at Moorfields Eye Hospital NHS Foundation Trust and UCL Institute of Ophthalmology, 162 City Road, ECV1 2PD London, UK; INSERM, CNRS, Institut de la Vision, Sorbonne Universités, UPMC Univ Paris 06, 17 rue Moreau, 75012 Paris, France

**Keywords:** Cystic macular lesions, Grading system, Optical coherence tomography, Usher syndrome, Genotype, Phenotype

## Abstract

**Background:**

To evaluate novel grading system used to quantify optical coherence tomography (OCT) scans for cystic macular lesions (CML) in Usher syndrome (USH) patients, focusing on CML associated alterations in *MOY7A* and *USH2A* mutations.

**Methods:**

Two readers evaluated 76 patients’ (mean age 42 ± 14 years) data prospectively uploaded on Eurush database. OCT was used to obtain high quality cross-sectional images through the fovea. The CML was graded as none, mild, moderate or severe, depending on the following features set: subretinal fluid without clearly detectable CML boundaries; central macular thickness; largest diameter of CML; calculated mean of all detectable CML; total number of detectable CML; retinal layers affected by CML. Intra-and inter-grader reproducibility was evaluated.

**Results:**

CML were observed in 37 % of USH eyes, while 45 % were observed in *MYO7A* and 29 % in *USH2A* cases. Of those with CML: 52 % had mild, 22 % had moderate and 26 % had severe changes, respectively. CML were found in following retinal layers: 50 % inner nuclear layer, 44 % outer nuclear layer, 6 % retinal ganglion cell layer. For the inter-grader repeatability analysis, agreements rates for CML were 97 % and kappa statistics was 0.91 (95 % CI 0.83-0.99). For the intra-grader analysis, agreement rates for CML were 98 %, while kappa statistics was 0.96 (95 % CI 0.92-0.99).

**Conclusions:**

The novel grading system is a reproducible tool for grading OCT images in USH complicated by CML, and potentially could be used for objective tracking of macular pathology in clinical therapy trials.

## Background

Usher syndrome (USH) is a rare autosomal recessive group of disorders characterized by retinitis pigmentosa (RP), sensorineural hearing loss and vestibular dysfunction. Three clinical types are distinguished (USH1, USH2 and USH3) and additional atypical subtypes have been described associated with 10 causative genes and three additional loci [1–5]. Hearing impairment is a first symptom to develop in USH. Visual symptoms manifest as retinitis pigmentosa (RP), a progressive retinal dystrophy, with rod and secondary cone photoreceptor dysfunction and ultimate loss. USH is a clinically and genetically heterogeneous disorder, making diagnosis and treatment challenging [6–9]. Previous studies categorized the morphological retinal findings of USH patients, showing that cystoid macula edema and/or cystic macular lesions (CML) are the most common complications in RP associated with USH (USH-RP) [10, 11]. CML has been reported to be present in 8-56 % of all USH and/or RP patients [12–19].

Morphologically CML is characterized as a focal or generalized retinal thickening due to accumulation of fluid within retinal layers, which may lead to the formation of a lamellar or full-thickness macular hole. Moreover, even non-significant cystoid changes might contribute to a progressive impairment of visual acuity in RP, likely due to the degenerative process affecting retinal layers [20]. The central macula thickness (CMT) is assumed to be a potential morphological end-point for evaluating safety and efficacy of a treatment modality, which is aimed primarily for improving macular photoreceptor cells associated structure and/or function. Thus, it is of a key importance to have a sophisticated grading system for quantification of CML in USH-RP associated with CML.

Even though CML is a frequent complication of RP and despite of several attempts to grade CML [21, 22], a need remains for an image grading protocol based on optical coherence tomography (OCT) to assist clinicians in describing all relevant aspects of an individual type of CML in USH-RP cases. We propose a new CML grading system for USH-RP patients, including comparative evaluation of CML between USH types and most frequent USH-associated genes, i.e. *MYO7A* and *USH2A*. Such a grading system would allow evaluation of USH-RP prognostic factors and treatment decisions in a very detailed way.

## Methods

### Subjects and database

This prospective observational cohort study was conducted according to the tenets of the Declaration of Helsinki and received authorization from Ile-de-France V ethical board committee for human research (June 4th 2013) and from the French regulation agency for medication (September 3rd 2013) (http://clinicaltrials.gov/ct2/show/NCT01954953). The Eurush database (www.eurush-database.org) was used where subjects’ data were uploaded on a prospective manner. The participants were recruited at the Centre Hospitalier National d’Ophtalmologie des Quinze-Vingts, Paris, France. The study procedure was fully explained to all patients, and their informed written consent was obtained. Seventy six patients with a clinical diagnosis of USH were enrolled (age 42 ± 14 years). The inclusion criteria were: clinical characteristics for USH1, USH2 and USH3 as defined by the Usher syndrome consortium [23]; informed consent and agreement to participate in the study. Exclusion criteria were: systemic pathologies or severe ocular pathologies; systemic or topical medication usage; otolaryngology pathologies which could contaminate the results; unwillingness to provide a blood sample for genetic test; unwilling and/or unable to undergo the study procedures. In more details we analyzed two groups, i.e. having mutations in genes *USH2A* and *MYO7A*.

Each patient underwent standard comprehensive ophthalmological examinations, which included assessment of the medical and family history, best corrected visual acuity (BCVA) using *Early Treatment Diabetic Retinopathy Study* (EDTRS), tonometry, slit-lamp biomicroscopy, direct and indirect ophthalmoscopy, static and kinetic perimetry using standard III4e isopters (Haag Streit, Koeniz, Switzerland). Full-field electroretinography (ERG) was performed with the Espion system (Espion Diagnosys, Littleton, MA), whereas multifocal electrophysiological recordings were carried out using the VERIS 4.9.1 system (Electro-Diagnostic Imaging, Inc., Redwood City). OCT and fundus autofluorescence (AF) were carried out using HRA II confocal scanning laser ophthalmoscope (Heidelberg Engineering, Heidelberg, Germany).

### Standard cystic macular lesions grading procedure

OCT image grading was performed in XV/XX reading centre (Paris, France). High quality macula scans were acquired using Spectralis HRA + OCT (Heidelberg Engineering, Dossenheim, Germany). The macular acquisition protocol consisted of 19 line raster fovea centered scans with at least a 15° by 15° region. The standard file transfer protocol was employed to transfer images in anonymous format to a local server. Five single scans lines, i.e. foveal scan, two below and two above the fovea were used for determining CML in both eyes. Both eyes were checked for CML. Only one (right) eye high quality cross-sectional image through the fovea was used for further grading. If CML were not present in foveal line scan but appeared in other 2 lines below or above the fovea, the closest line to the fovea was used for grading.

The trained graders performed OCT image grading using a novel grading system for CML quantification. In addition, 3 randomly selected tomograms were provided to each grader to test reproducibility. All reproducibility testing were performed 5 weeks after initial grading to avoid possible reader recall of the initial arbitration and to monitor temporal variability. The intra-grader and inter-grader reproducibility was calculated, in details described elsewhere [24]. Scan quality was estimated depending on presence/absence following features: fovea non determinable on OCT, incorrect positioning, and very poor saturation due to cataract and/or vitreous strands and opacities.

**Fig. 1:**
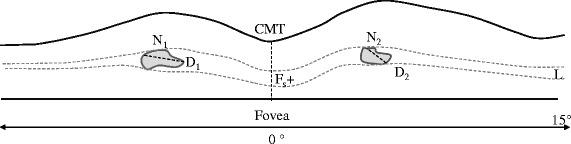
The schematic grading of cystic macular lesions in optical coherence tomography images. Retinal cystic lesions were quantified through the fovea line scan within to 15° eccentricity. Evaluated parameters were: total quantity of cystic macular lesions (N = N1 + N2 + […] + Nn); averaged largest diameter of all cystic lesions (D = (D1 + D2 + […] + Dn)/n); layer affected by cystic lesions (L); presence/absence of foveal retinal layer/-s swelling (Fs+/ Fs-), if boundaries of cystic lesions were not detectable (e.g. media opacities); central macular thickness (CMT)

The protocol used to define the CML included the presence of cystoids spaces defined as hypo-reflective zone visible on at least two views of the sequential line scans in macula area. The boundaries of each CML were manually marked using Adobe Photoshop CS5.5 version. A grading protocol was defined covering all possible morphological alterations and characteristic patterns typically associated with CML, namely: 1. Subretinal fluid without clearly detectable CML boundaries (F_s_); 2. CMT; 3. Largest diameter of CML (D_n_); 4. Calculated mean of all detectable CML (D); 5. Total number of detectable CML (N); 6. Retinal layers affected by CML (L) (Fig. 1). Hence, CML was graded as mild, moderate or severe depending on above described features pattern (Table 1).

**Table 1:**
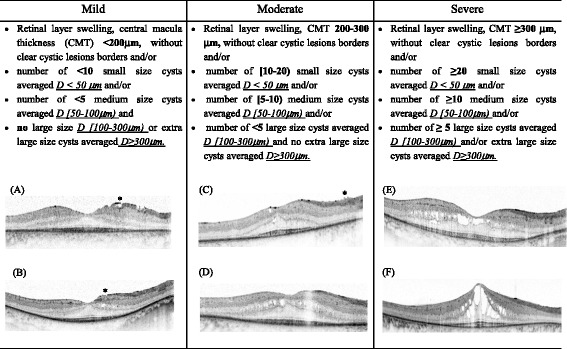
The grading system for the cystic macular lesions severity and selected tomograms examples in Usher syndrome patients. *A*-*B Mild*: the right eye of 26 year male with mutation in *USH2A*:c.7595-3C > G+ c.7595-2144A > G, having less than 10 small size cystic lesions and central macula thickness (CMT) within 200 μm (A), the right eye of 38 year male with mutation in *USH2A*: *E767fs*, having less than 10 medium size cystic lesions (B); *C*-*D Moderate*: the right eye of 14 year female with mutation in *MYO7A*: *L2186P* *2, having more than 10 medium size cystic lesions and CMT up to 300 μm (C), the right eye of 31 year male with mutation in *USH2A*:*T4337M* + *C717G*, having up to 20 small and up to 10 medium size cystic lesions and one large diameter cyst (D); *E*-*F severe*: the right eye of 35 year female with mutation in *USH2A*:*V218E* + *G660R*, having more than 10 medium size cystic lesions, more than 5 large and several extra large (E), the right eye of 24 year male with mutation in *MYO7A*:N1182K*2, having several extra large diameter cystic lesion (F). * The presence of epiretinal membrane

Currently, there is no available standard CML grading system and CMT is used for edema evaluation. Only for comparative reasons with the literature data, we extinguished CMT criterion from CML grading system for the more detailed analysis. Once the grading database was verified and closed, the grading and clinical data were merged for the analysis.

### Statistical analysis

Statistical analysis was carried out using SPSS software (version 19.0, SPSS Inc., Chicago, USA). We analyzed CML in the whole cohort and in two groups associated with mutations in *USH2A* and *MYO7A*. Pearson correlation (r) was used to assess the correlation between central macular thickness and age in a whole cohort and associated genes, namely *USH2A* and *MYO7A*. One-way analysis of variance (ANOVA) was performed to detect differences between CML severity in associated gene *USH2A* and *MYO7A*. The percent agreement and the kappa statistics were calculated as measures for categorical variables (quality of scan and presence of CML). Percent agreement was computed as the number of concordant grading pairs divided by the total number of grading pairs multiplied by 100. All numerical data are presented here as arithmetic means ± standard deviation (SD). A two side P value less than 0.05 was considered statistically significant.

## Results

### Main clinical characteristics

Our cohort consisted of 76 patients, 41 (54 %) were males. All 76 (100 %) patients were found to carry at least one mutation, while 68/76 (89 %) of them carrying two mutations in USH-associated genes. The methodology used to detect mutations (complete exon sequencing) in USH associated genes is described in details elsewhere [2]. The prevalence of mutations in USH-associated genes was as following: 13/76 (17 %) *MYO7A*, 4/76 (5 %) *CDH23*, 2/76 (3 %) *PCDH15*, 1/76 (1 %) *USH1C*, 44/76 (58 %) *USH2A*, 8/76 (11 %) *VLGR1*, 3/76 (4 %) *CLRN1*, and 1/76 (1 %) had mutations in *VLGR1* and *MYO7A* genes. The mean age was of 42 ± 14 years (range of 14 to 74 years). There was no statistically significant difference in age between *MYO7A* and *USH2A*: 41 ± 15 (14 to 69) years for *MYO7A* and 44 ± 13 (23 to 74) years for *USH2A*. The average BCVA for the whole cohort was 0.40 logMAR units (range,−0.1 to 1.5, Snellen equivalent of 20/50), while 0.61 logMAR units (range 0.1 to 1.5, Snellen equivalent of 20/80) for *MYO7A* and 0.34 logMAR units (−0.1 to 1.5, Snellen equivalent of 20/40) for USH2A. *MYO7A* patients tended to have worse BCVA compared to *USH2A*, although the difference did not reach statistically significant level (*p* > 0.05).

### Grading of optical coherence tomography scans

**Fig. 2:**
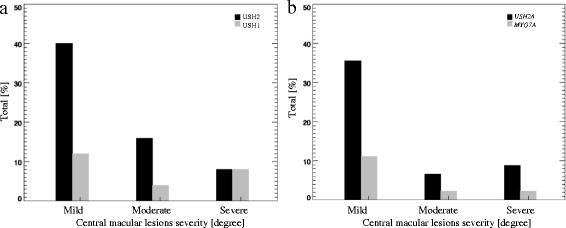
The prevalence of cystic macular lesions severity in Usher syndrome cases. The prevalence of cystic macular lesions in USH 1 and USH 2 **a**, the prevalence of cystic macular lesions in *USH2A* and *MYO7A* **b**

The OCT scans were graded using the novel CML grading system (Table 1). Of the 76 USH patients, for 13 patients OCTs were not performed due to the advanced stages of USH affecting the ocular media: 2/13 (15 %) in *MYO7A*, 6/13 (46 %) in *USH2A*, and 5/13 (39 %) in other genes. Of those with performed OCT, CML were present in 23/63 (37 %) cases. CML were found in 12/23 (52 %) males and 11/23 (48 %) females, without any evident gender preponderance. We observed that USH1 (and *MYO7A*) tend to have higher incidence of CML compared to USH2 (and *USH2A*): 6/14 (43 %) in USH1 (and 5/11 (45 %) in *MYO7A*) and 14/45 (31 %) in USH2 (and 11/38 (29 %) in *USH2A*), respectively. The CML severity for a USH cohort was as following: 12/23 (52 %) mild, 5/23 (22 %) moderate, and 6/23 (26 %) severe. USH1 (and *MYO7A*) tend to have more severe CML, while USH2 (and *USH2A*) tend to have mild CML (Fig. 2a and b).

**Fig. 3:**
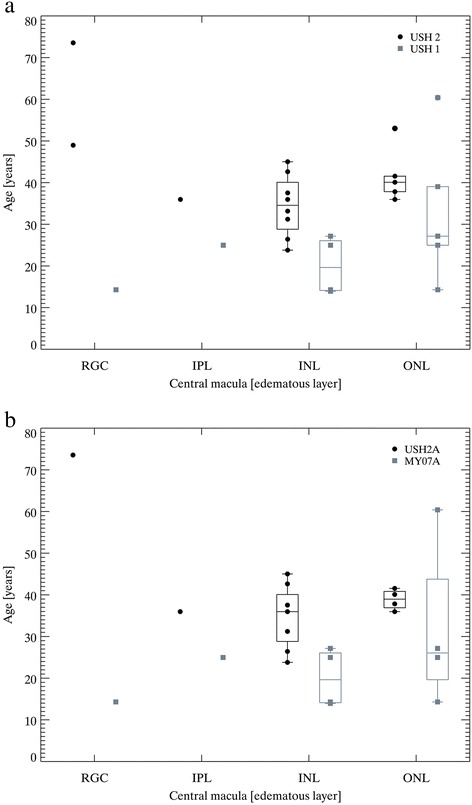
Cystic macular lesions distribution by retinal layers distribution in Usher syndrome cases. The prevalence of CML by affected retinal layers in USH1 vs USH2 **a**, the prevalence of CML by affected retinal layers in *USH2A* vs *MYO7A* **b**

Analyzing retinal layers integrity, CML tended to affect one retinal layer: 16/23 (70 %) monolayer vs 7/23 (30 %) poly-layer (*p* < 0.05). Of those with monolayer involvement, the inner nuclear layer (INL) was affected in 8/16 (50 %), outer nuclear layer (ONL) in 7/16 (44 %), and the retinal ganglion cell layer (RGC) 1/16 (6 %) cases. Of those with poly-layer involvement, the INL was affected in 6/7 (86 %), ONL in 7/7 (100 %), RGC 2/7 (29 %) and the inner plexiform layer (IPL) was affected in 2/7 (29 %) cases. There was no statistically significant difference in CML retinal layers predisposition between USH types or USH-associated genes (Fig. 3a and b). When study cohort was divided in four groups, those with monolayer CML, i.e. in RGC, INL, ONL, and poly-layer CML, the mean CMT was as following: 176 μm (RGC), 218 ± 57 μm (INL), 166 ± 52 μm (ONL), and 332 ± 106 μm (poly-layer), respectively. The more severe CML were observed in cases affecting INL compared to other layers (*p* < 0.001).

**Fig. 4:**
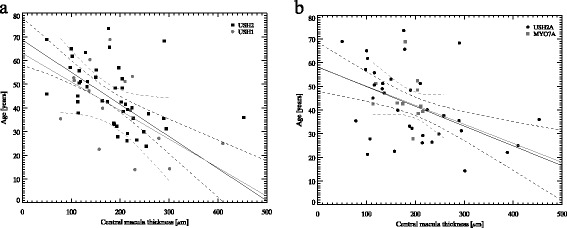
The linear regression analysis between age and central macula thickness in all Usher cohort. The statistically significant negative association was observed between CMT and patient age both in USH1 and USH2: *r* = −0.51 and *r* = −0.47 **a**; and as following in *USH2A* and *MYO7A*: *r* = −0.58 and *r* = −0.44 **b**

CMT values tend to be higher in *MYO7A* group compared to *USH2A*: 263 ± 91 μm and 212 ± 89 μm, respectively, however this was not statistically significant. Linear regression analysis showed a statistically significant association between CMT and age (at the time of examination) in USH1 and USH2 (*r* = −0.51, *p* = 0.01 and *r* = −0.47, *p* = 0.01) and as following *USH2A* and *MYO7A* (*r* = −0.58, *p* = 0.01 and *r* = −0.44, *p* = 0.01). (Fig. 4a and b).

### Quality assurance data for a novel grading system

The evaluation of the proposed system is done using Eurush database estimating intra-grader and inter-grader repeatability. The time to grade one tomogram with CML required in averaged 5 min ± 1 min for the first and 6 min ± 1 min for the second grader (not statistically significant *p* > 0.05). The difference in mean CML diameter was 2 μm ± 1.5 μm between the two graders performed grading of OCT scans. Reproducibility rates were high for the categorical variables scan quality at the fovea and CML. For the inter-grader repeatability analysis, agreements rates were 97 % (95 % CI 0.915-1.00) and 95 % (95 % CI 0.89-1.00) for the scan quality at the fovea and CML, respectively. The kappa statistics was 0.93 (95 % CI 0.85-1.00) and 0.91 (95 % CI 0.83-0.99) for the scan quality at the fovea and CML, respectively. For the intra-grader analysis, agreement rates were 96 % (95 % CI 0.80-1.00) and 98 % (95 % CI 0.89-1.00) for the scan quality at the fovea and CML, respectively. Likewise, kappa statistics was 0.91 (95 % CI 0.80-0.95) and 0.96 (95 % CI 0.92-0.99) for the scan quality at the fovea and CML, respectively.

## Discussion

In the present study, we established a novel grading system that showed to be reliable and reproducible in our reading centre for evaluating CML in USH-RP patients. Our system consists of CML detection assessed by trained graders, followed by evaluation of CML severity and assessment of affected retinal layers. Using the novel grading system OCT scans can be graded in a standard manner in about 5 minutes.

To the best of our knowledge, there are limited references in the literature regarding how to evaluate CML severity in USH-RP cases. We found that using our standard method, CML quantification gives a detailed view on retinal morphological changes which is crucial for disease progression monitoring and exact end-points estimation. In addition, the incidence of CML was compared within the most frequent USH-associated genes, i.e. *MYO7A* and *USH2A*. In our cohort of 76 USH patients, CML rate was found to be 37 %, while in *MYO7A* was of 45 % and *USH2A* was of 29 %. Almost half of *MYO7A* cases were associated with CML, although a statistically significant difference between *MYO7A* and *USH2A* groups was not reached. In addition, *MYO7A* cases tend to represent by severe CML, while *USH2A* tend to have mild CML. So far no pathophysiological studies were performed analyzing CML incidence differences in *USH2A* vs *MYO7A*. Thus, it definitely requires more detailed investigations.

Our findings of 37 % having CML in OCT scans are comparable to Hajali et al’s, namely 35 % USH2 and 39 % autosomal recessive RP patients had CML in OCT scans [25, 26]. It must be acknowledged that there is a great difference in the incidence of CML among the various cohorts of RP patients, ranging from 8-15 % [12, 16, 17] to 25-56 % [14, 15, 19, 25]. One reason might be that ascertaining CML might vary due to race and ethnicity differences. CML incidence rates are highly dependent on RP inheritance pattern (recessive, dominant or X linked) and so genotypic preponderance can also influence the results [11]. Some studies were very small which can also lead to unstable estimates. Finally, the lack of structured grading might also make ascertaining CML difficult and unreliable.

In the current study we showed that eyes with CML have 30 % greater CMT (235 ± 98 μm) compared to those without CML (164 ± 59 μm). Gorovoy et al. [20] also reported similar findings: 28 % of RP patients had CML and half of them had increased central foveal thickness (239 ± 10 μm). Some differences could be explained by differences in age between studies and the variability in disease stage, in particularly the presence of retinal atrophic changes. Our study clearly showed that younger patients tended to have higher rates of CML (*r* = −0.47, *p* = 0.01). In a large enough cohort, multiple regression analysis where CML severity could be tested against age and CMT would potentially provide new insight into the natural disease history.

Additionally, we showed that CML tended to be present in INL (50 %) and ONL (44 %). Makiyama et al. [18] showed 27 % incidence of CML, while cystic lesions were observed in the same layers as in our study, namely INL (99 %) and ONL (28 %) and a minority in RGC. Fakin et al. [19] observed 56 % CML in USH-RP patients, while CML were distributed in INL (60 %), INL and ONL (33 %) and OPL (7 %). To the best of our knowledge, there was no pathophysiological studies performed so far analyzing CML affinity for the particular retinal layers.

The natural course of CML associated with RP is incompletely understood with several theories proposed. Heckenlively et al. [26] proposed that CML might be a result of an autoimmune process. The high incidence of CML in INL and ONL could be explained by microglial cells activation as a result of the autoimmune process [27 - 29]. An alternative hypothesis could be that CML is a result of a breakdown in the retinal barrier at the level of the retinal pigment epithelium which leads to a fluid leakage in the macula. The breakdown of blood-retinal barrier potentially results in accumulation of plasma proteins which exert a high oncotic pressure in the neural interstitium, which tends to produce interstitial edema [30–32]. It is possible that both mechanisms occur together [33, 34]. Lastly, it is possible that the “cystic spaces” are actually due to apoptosis and therefore simply represent a space left from tissue loss [35].

## Conclusion

A standard OCT-based retinal image evaluation in USH-RP represents an urgent need both for clinicians and scientists and calls for a reading centers focused of OCT evaluation. The proposed grading system supplies a reproducible tool for CML quantification in USH-RP, and eventually could be used for objective tracking of macular pathology in clinical trials. Using our grading system we observed that CML is a common retinal complication in USH-RP and showed that CML tended to be present in both inner and outer nuclear layer. Moreover, we found that *MYO7A* cases tend to have a higher CML incidence rate and more severe CML compared to *USH2A*.

We describe for the first time a novel grading system for CML evaluation that is based on expert knowledge and requires trained personnel. In the future, the novel grading system might be applied using semi-automatic image analysis for CML severity grading, taking into account functional and/or other significant structural measures [36–40]. The system has a potential to be implemented in reading centers as a sophisticated tool for retinal structure evaluation in USH-RP. Surely, it would be directly applicable to any CML in other retinal diseases (e.g. diabetic retinopathy, age related macula edema) and aid treatment challenges in other rare retinal degenerations.

## Abbreviations

CML: cystic macular lesions
OCT: optical coherence tomography
RP: retinitis pigmentosa
USH: usher syndrome
USH-RP: retinitis pigmentosa associated with USH
